# Hemoglobin levels and blood transfusion in patients with sepsis in Internal Medicine Departments

**DOI:** 10.1186/s12879-016-1882-7

**Published:** 2016-10-13

**Authors:** Gassan Fuad Muady, Haim Bitterman, Arie Laor, Moshe Vardi, Vitally Urin, Nesrin Ghanem-Zoubi

**Affiliations:** 1 Internal Medicine Department, Carmel Medical Center, Michal 7, 34362 Haifa, Israel; 2 The Ruth and Bruce Rappaport Faculty of Medicine, Technion - Israel Institute of Technology, Haifa, Israel; 3 Harvard Clinical Research Institute, Boston, MA USA; 4 School of Public Health, Boston University, Boston, MA USA; 5 Infectious Diseases Unit, Rambam Health Care Campus, Haifa, Israel

**Keywords:** Sepsis, Anemia, Blood transfusion, Internal medicine units, Hemoglobin

## Abstract

**Background:**

Acute reduction in hemoglobin levels is frequently seen during sepsis. Previous studies have focused on the management of anemia in patients with septic shock admitted to intensive care units (ICU’s), including aggressive blood transfusion aiming to enhance tissue oxygenation.

**Aim:**

To study the changes in hemoglobin concentrations during the first week of sepsis in the setting of Internal Medicine (IM) units, and their correlation to survival.

**Design:**

Observational prospective study.

**Methods:**

We recorded hemoglobin values upon admission and throughout the first week of hospital stay in a consecutive cohort of septic patients admitted to IM units at a community hospital, the patients were enrolled into a prospective registry.

Data on blood transfusions was also collected, we examined the correlation between hemoglobin concentrations during the first week of sepsis and survival, the effect of blood transfusion was also assessed.

**Results:**

Eight hundred and fifteen patients (815) with sepsis were enrolled between February 2008 to January 2009. More than 20 % of them had hemoglobin levels less than 10g/dL on admission, a rate that was doubled during the first week of sepsis. Overall, 68 (8.3 %) received blood transfusions, 14 of them (20.6 %) due to bleeding. Typically, blood transfusion was given to older patients with a higher rate of malignancy and lower hemoglobin levels. While hemoglobin concentration on admission had strong correlation with in-hospital mortality (O.R-0.83 [95 % C.I. 0.74–0.92], blood transfusion was not found to be an independent predicting factor for mortality.

**Conclusion:**

Anemia is very common in sepsis. While hemoglobin level on admission exhibit independent correlation with survival, blood transfusion do not.

## Background

Anemia is a common problem encountered in patients with sepsis. Several mechanisms contribute to acute reduction in hemoglobin levels in the setting of sepsis, including reduced production of red blood cells induced by the systemic inflammatory response, as well as increased destruction of red cells due to hemolysis and bleeding. The combination of anemia and alterations in oxygen consumption induced by sepsis may augment impairment of tissue oxygenation. Treatment of septic patients also aims at optimizing the delivery of oxygen to tissues in order to alleviate cellular hypoxia and the progression of cell dysfunction, in an attempt to avoid or attenuate the development of multiple organ dysfunction syndrome [1].

Based on this notion, maintenance of adequate blood hemoglobin level was suggested as one of the tools to diminish sepsis induced tissue damage. The concept of maintaining a minimal hematocrit level in the acute treatment of sepsis was emphasized in the early goal-directed therapy (EGDT) trial, by targeting a hematocrit value of 30 % in patients with low ScvO2 (Central venous oxygen saturation) during the first 6 h of the resuscitation of septic shock [2].

Of note, recent trials found no benefit for protocol-based resuscitation in the context of septic shock [3–5].

According to the Surviving Sepsis Campaign guidelines updated in 2012, septic patients with hemoglobin concentration of <7g/dL should receive RBC transfusion in order to achieve hemoglobin target values of 7–9gr/dL [6].

This recommendation is based on the Transfusion Requirements in Critical Care trial [7], in which less than 5 % of the enrolled patients were with primary diagnosis of sepsis, while the majority endured respiratory or cardiovascular diseases or trauma. Furthermore, the study population was significantly younger with a mean age of 57.6 years [7], while more commonly the mean age of septic patients is significantly older [8, 9], and the majority of septic patients in previous studies were treated in general departments rather than in ICUs [8, 10]. Furthermore, in recent studies targeting the issue of optimal hemoglobin level in sepsis, the focus was on patients with septic shock that were treated in ICU settings.

Worldwide, steadily increasing numbers of elderly patients with sepsis are treated in general departments including Internal Medicine units by internist doctors. Only few studies focus directly on this group of patients. In the present study, we characterized acute changes in hemoglobin levels during the first week of sepsis in patients admitted to IM (Internal Medicine) departments and its correlation to in-hospital mortality and overall survival. In order to evaluate the practice of blood transfusion in this setting, we also compared the characteristics and outcome of patients who received blood transfusions as part of their treatment to those who did not.

## Methods

Our study is based on a registry of septic patients admitted to IM departments during one- year period in a 450-bed community-based university hospital in Haifa, Israel.

The data were collected prospectively for patients fulfilling sepsis criteria on their admission according to the American College of Chest Physicians/Society of Critical Care (ACCP/SCCM) Consensus Conference in 1991 [11], i.e. patients who were admitted with suspected infection and at least two of the criteria for SIRS. No exclusion criteria were employed.

The patients were categorized with sepsis based on the above- mentioned criteria determined by the treating physicians. The definitions were stated in the electronic questionnaire where the admitting physician marked the appropriate category of sepsis for the patient upon admission only. The diagnosis was not revised any more later on the hospital stay or during the discharge. During the study period, the hospital had no specific constructed protocol for the diagnosis and treatment of sepsis. Yet, generally the septic patients treatment was affected largely by the concept of starting antibiotics and fluids as soon as possible once the patient was diagnosed with sepsis.

The data that were recorded included information related to current illness such as vital signs, e.g. heart rate, respiratory rate, body temperature, systolic and diastolic blood pressure, and oxygen saturation. Physicians were instructed to register the worst value of each of the variables until admission to the department, including Emergency Department values. Demographic data that were collected included gender, age, functional status and mental status. Data on co-morbidities included ischemic heart disease, congestive heart failure, chronic obstructive pulmonary disease, hypertension, diabetes mellitus, chronic kidney disease, liver disease and cancer. Clinical status at presentation was ascertained using ACCP/SCCM sepsis definition (sepsis, severe sepsis, septic shock) [11].

Laboratory results were drawn automatically and included hemoglobin concentrations on admission, the minimal values during the first 7 days of sepsis and values at the end of the first week. We also collected data on blood transfusions during hospitalization.

Major outcomes were collected including mortality (in-hospital and after discharge) up to 2 years of follow up.

The study was approved by the hospital local ethics committee: Carmel medical center ethics committee.

No informed consents were needed from the patients.

## Statistical methods

Baseline characteristics were compared with the ANOVA (F-Test) and *t*-test for continuous variables (3 or 2 categories, respectively), and Wilcoxon’s scores or chi square test for parametric or discrete variables, respectively.

Univariate comparison of survival between two or more groups with the LIFETEST procedure, which computes nonparametric estimates of the survival distribution function. We used the product-limit (Kaplan and Meier). PROC LIFETEST computes nonparametric tests to compare the survival curves of two or more groups. The procedure also computed rank tests of association of the survival time variable with other concomitant variables.

Survival models (explaining survival by multiple continuous and discrete variables), used the partial likelihood of the Cox model as the likelihood and generated a chain of posterior distribution samples by the Gibbs Sampler. Summary statistics, convergence diagnostics, and diagnostic plots were provided for each parameter. A backward stepwise elimination method using Wald Chi-Square was used to achieve a model with only significant factors.

Logistic regression was used to explain two level parameters such as hospital mortality. The computations were done by SAS9.2 Software procedures GLM, FREQ, NONPAR1WAY, LOGISTIC, PHREG, LIFETEST and more.

## Results

Between February 1, 2008 and January 30, 2009, 815 patients were admitted to the IM departments at our medical center with a diagnosis compatible with sepsis. We classified our cohort according to hemoglobin levels as follows: normal to mildly reduced hemoglobin concentration (≥10g/dL), moderately reduced concentration (≥7 and <10g/dL) and severely reduced hemoglobin concentration (<7g/dL) . The characteristics of the cohort are detailed in Table 1. As expected in this setting the study population was old with a high proportion of patients with cognitive and functional status decline and co-morbidities. Most patients (77 %) had hemoglobin concentrations of ≥ 10g/dL on admission, while less than 1 % had severely reduced levels. The average values of mean corpuscular volume (MCV) and mean corpuscular hemoglobin (MCH) on admission for the entire cohort were 85.7 ± 6.6 (normal range 80–96 fl) and 28.68 ± 4.38 (normal range 27.5–33.2 pg), respectively and did not differ significantly between the three groups classified by hemoglobin concentration on admission. As shown it Table 1 the groups of patients with moderately and severely reduced hemoglobin levels on admission had higher proportions of chronic kidney disease (CKD) and dialysis and terminal diseases (expected life expectancy of less than one month) and/or malignancy than the group of normal to mildly reduced hemoglobin concentration. The latter group also had a significantly higher systolic blood pressure and lower serum creatinine and urea. There were no significant differences between the three groups concerning the rates of severe sepsis and septic shock on presentation, as well as in respiratory parameters, need for respiratory support, and acute cardiovascular events (Table 1).

**Table 1:** Characteristics of patients according to hemoglobin levels on admission, classified into three groups: HGB ≥ 10, HGB ≥ 7- < 10, HGB < 7 g/dL

Parameter	HGB ≥ 10	HGB ≥ 7- < 10	HGB < 7	*P value*
no. of patients	631 (77 %)	177(22 %)	7(1 %)	
Age (Mean ± S.D.)	74.3 ± 16.2	77.3 ± 13.5	72.5 ± 15.1	0.07
Females (%)	308 (48.8 %)	83(46.8 %)	5(71.4 %)	0.97
Residence				
Home	502(79.6)	138(78.0)	5(71.4)	
Nursing home/LTCF	129(20.4)	39 (22)	2(28.6)	0.50
Mental status at baseline				
Dementia	208 (36.1)	70(43.7)	2(28.5)	0.18
Normal	363 (63)	88 (55)		
Other	5 (0.8)	2 (1.2)	5 (71.4)0 (0)	
Basic functional status				
Debilitated	250(40.1)	80(45.9)	3(42.8)	0.06
Partially debilitated	62(9.9)	22(12.6)	2(28.5)	
Normal	310(49.8)	72(41.3)	2(28.5)	
CHF	211(33.4)	68(38.4)	2(28.5)	0.30
IHD	255(40.4)	76(42.9)	1(14.2)	0.97
Diabetes mellitus	217(34.3)	70(39.5)	1(14.2)	0.45
CKD	158(25.0)	69(38.9)	2(28.5)	0.0007
Chronic dialysis	11(1.7)	10(5.6)	0(0)	0.011
Active cancer	97(15.3)	71(40.1)	2(28.5)	<.0001
Terminal disease	42(6.6)	37(20.9)	2(28.5)	<.0001
Sepsis stage on admission				
Severe sepsis	57(9.0)	17(9.6)	0(0)	
Septic shock	28(4.4)	13(7.3)	2(28.5)	0.18
Source of infection				
Pneumonia	263(41.6)	54(30.5)	2 (28.5)	
UTI	183(29.0)	51(28.8)	1(14.2)	0.87
SSTI	34(5.3)	18(10.1)	1(14.9)	
Intra-abdominal	6(3.3)	6(3.3)	0(0)	
Other	37(5.8)	18(10.1)	1(14.2)	
Unknown	105(16.6)	30(16.9)	2(28.5)	
Acute myocardial infarction	23(3.65)	7(3.9)	1(14.3)	0.34
Acute respiratory failure	9(1.4)	1(0.5)	0(0)	0.33
CHF exacerbation	25(3.9)	6(3.3)	0(0)	0.60
Systolic BP	121.9 ± 26.8	115.8 ± 27.7	111.2 ± 22.1	0.02
Glasgow coma scale	12.4 ± 3.9	12.1 ± 3.9	11.1 ± 5.1	0.49
Serum Creatinine	1.2 ± 0.9	1.6 ± 1.3	1.5 ± 1.1	0.0002
Serum urea	65.3 ± 46.3	82.8 ± 51.1	80.0 ± 51.4	***<.0001***

*HGB* hemoglobin, *CHF* congestive heart failure, *IHD* ischemic heart disease, *CKD* chronic kidney disease, *UTI* urinary tract infection, *SSTI* skin and soft tissue infection, *BP* blood pressure

**Fig. 1:**
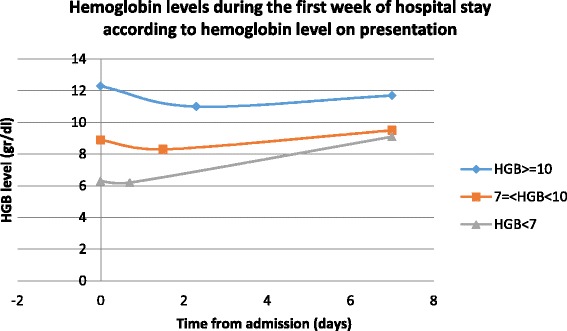
Hemoglobin level changes during first week classified according to admission level

Hemoglobin changes during the first week of hospitalization are shown in Fig. 1. On admission, the mean hemoglobin value of the whole study group was 11.5 ± 2.0g/dl. The maximal reduction in hemoglobin was seen within a mean time of 2.1 ± 1.9 days, with a mean decrease of 1.08 ± 1.04 g/dL. The absolute reduction was higher in patients with normal to mildly reduced hemoglobin concentration on admission compared to patients with moderately and severely reduced levels (1.2 ± 1.0, 0.5 ± 0.6 and 0.14 ± 0.25, respectively) and occurred later (2.3 ± 1.9 vs. 1.5 ± 1.8 and 0.7 ± 1.4 days, *P* < 0.001).

Lowest hemoglobin values throughout the first week were moderately reduced in 315 patients (38.7 %) and severely reduced in 18 (2.2 %). At the end of the first week, moderately and severely reduced levels were observed in 196 (24 %) and 4 (0.5 %) of the patients, respectively.

Sixty-eight patients (8.3 %) were transfused with packed red blood cells, the mean hemoglobin level which necessitate transfusion was 7.86 ± 1.16 gr/dl; the nadir hemoglobin levels in this group of patients occurred within a mean time of 4.4 ± 5.1 days from admission. Bleeding occurred in 22 patients, mostly gastrointestinal bleeding (63 %). Retroperitoneal, cranial, muco-cutaneous and genitourinary bleeding constituted the remaining events (37 %).

During the study, there was no specific transfusion protocol. Ordering blood transfusion is an independent decision of the treating physician based usually on clinical consideration rather the absolute levels of Hemoglobin.

Transfusion reactions occurred in two cases, and both were nonfatal hemolytic reactions. Table 2 shows comparison between patients who received blood transfusions to those who did not. Patients who received transfusions were older, with higher proportion of dementia, malignancy and terminal diseases. There were no significant differences in the rates of acute cardiovascular events or respiratory failure among patients who received transfusion and those who did not.

**Table 2:** Characteristics of patients who received blood transfusions and those who did not

Parameter	Transfused	Not transfused	*P* value
no. of patients	68	747	
Age (Mean ± S.D.)	78.8 ± 11.0	74.6 ± 16.0	0.03
Female n (%)	36(52.9)	360(48.1)	0.45
CHF	21(30.8)	260(34.8)	0.51
IHD	24(35.2)	308(41.2)	0.34
Diabetes mellitus	28(41.1)	260(34.8)	0.29
CKD	24(35.2)	205(27.4)	0.16
Chronic dialysis	2(2.9)	19(2.5)	0.84
Active cancer	23(33.8)	147(19.6)	0.006
Terminal disease	13(19.1)	68(9.1)	0.0083
Sepsis stage on admission			
Severe sepsis	6(8.8)	68(9.1)	0.66
Septic shock	6(8.8)	37(4.9)	
acute myocardial infarction	2(2.9)	29(3.9)	0.69
Acute respiratory failure	1(1.4)	9(1.2)	0.84
CHF exacerbation	4(5.8)	27(3.6)	0.34
HGB on admission	9.25 ± 1.91	11.73 ± 1.92	<0.0001
Minimal HGB	7.86 ± 1.16	10.68 ± 1.77	<0.0001
HGB on day 7	9.72 ± 1.36	11.38 ± 1.79	<.0001
Time from admission to minimal HGB (days)	2.30 ± 1.36	2.18 ± 1.79	0.9821
ΔHGB (admission-minimal HGB);	1.39 ± 1.44	1.06 ± 0.99	0.0127

*HGB* hemoglobin, *LTCF* long term care fascility, *CHF* congestive heart failure, *IHD* ischemic heart disease, *CKD* chronic kidney

**Fig. 2:**
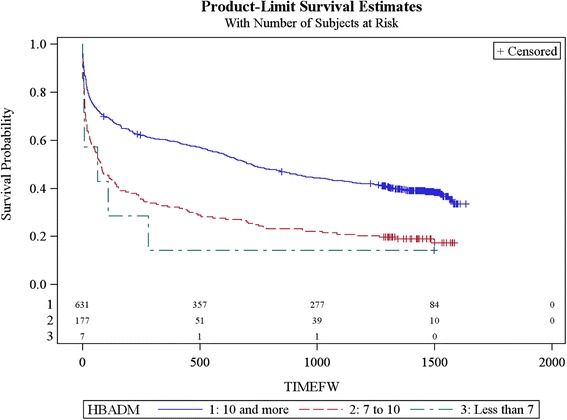
Survival probability according to hemoglobin level on admission

Thirty-four percent of the study cohort survived throughout a mean follow-up of 675.1 ± 634.8 days. The survival rate differed significantly according to hemoglobin concentration on admission as follows; 38.5 % in patients with normal to mildly reduced level, 18.6 % for moderately reduced level, and 14.2 % for severely reduced level (*p < 0.0001*), respectively. Kaplan-Meier curves of survival according to hemoglobin concentration on admission are given in Fig. 2. The same trend of survival is seen in comparisons that were based on the lowest value of hemoglobin during, and at the end of the first hospitalization week.

The in-hospital mortality rate was 22.8 % for the entire cohort with significantly increasing rates as hemoglobin admission level decreased.

The significant factors found to correlate with in-hospital mortality are summarized in Table 3. In adittion to other parameters, higher hemoglobin on admission was found to have a protective effect, OR = 0.83 (*P = 0.0004).*

**Table 3:** Odds ratios and Wald confidence intervals for in-hospital mortality

Parameter		O.R.	95 % C.I.	*P* value
Age	For increase of 1 year	1.020	1.001–1.040	0.0438
Terminal disease	Yes vs. no	1.964	1.097–3.517	0.0231
Stand without help	No vs. yes	1.869	1.153–3.027	0.0111
Dyspnea on admission	No vs. yes	0.339	0.223–0.517	<0.0001
Abnormal ECG on admission	No vs. yes	0.553	0.370–0.829	0.0041
Systolic blood pressure on admission	For increase of 1 mmHg	0.992	0.985–0.999	0.0290
Glasgow coma scale on admission	For increase of 1 point	0.902	0.858–0.947	<.0001
Hemoglobin on admission	For increase of 1 gr/dl	0.830	0.748–0.921	0.0004
Serum urea on admission	For increase of 1 mg/dl	1.010	1.006–1.014	<.0001

*O.R* odds ratio, *CI* confidence interval, *ECG* electrocardiograph, *BP* blood pressure

Although a higher rate of in-hospital mortality (36.7 % vs. 21.5 % *p* < 0.05) and a lower survival rate (17.6 % vs. 35.4 % *p* < 0.05) were seen in patients who received blood transfusions, this parameter was not found to be an independent significant factor in multivariate regression and survival model analyses.

## Discussion

The present study shows that patients with sepsis admitted to IM departments frequently have anemia at an early stage of sepsis, which worsens during the first few days after admission to the hospital. The mean hemoglobin concentration on admission in our cohort was 11.5 ± 2.0. A hemoglobin concentration of less than 10g/dL was observed in more than 20 % of the present study patients already on presentation. This rate increased to almost 40 % during the first few days of hospital stay, and still observed in one fourth of our septic patients at the end of first week. In the absence of information on baseline hemoglobin values before admission, the low levels on presentation may either be an expression of chronic anemia in patients with a high rate of co-morbidities or an acute reduction due to sepsis, as well as a combination of both etiologies. Anemia in sepsis is observed frequently [12, 13] and has a multifactorial etiology, including a decrease in production of erythropoietin due to inflammatory cytokines release, such as tumor necrosis factor (TNF-α), interleukin-1 (IL-1), and interleukin-6 (IL-6). Interferon gamma and IL-1 also seem to act directly by inhibiting the growth of pro-erythrogenic cells and apoptosis of erythroid precursors, thus contributing to the development of anemia in these patients [14]. Other mechanisms that may contribute to hemoglobin reduction in sepsis are stress-induced gastrointestinal bleeding/hemodilution due to fluid overload, recurrent blood withdrawal for laboratory analysis, impaired iron metabolism, hemolysis as part of the pathogenesis of some infectious processes, bleeding due to DIC, and possibly increased red cell destruction due to alterations in red blood cells membranes [14].

As mentioned previously, another possible explanation for low hemoglobin levels on presentation in our cohort maybe due to anemia of chronic diseases. The study population had a high rate of co-morbidities, including malignancy and CKD that were documented in more than half of the cases. CKD and active cancer are well known causes of anemia due to several mechanisms [15, 16]. On the other hand, the worsening in hemoglobin levels in our study subjects during the first week of hospital stay presented in an average reduction of 1.09 ± 1.04 g/dL with minimal concentrations occurring within 2.19 ± 1.99 days from presentation reflects development of anemia as an important part of the course of sepsis..

The fact that the absolute reduction was higher in patients with higher hemoglobin concentrations on admission (normal to mildly reduced vs. moderately reduced vs. severely reduced levels) and occurred later (Fig. 1) may reflect their presentation for treatment at an earlier stage of sepsis and could explain its correlation with better survival (Fig. 2). In logistic regression analysis, hemoglobin levels on admission, had a correlation with in hospital mortality (OR. 0.83, *p* = 0.0004) the higher the hemoglobin level the better was the prognosis. The negative correlation between low hemoglobin concentration and survival in sepsis had been observed in previous studies [12, 13].

The fact that the present study is an observational one, supplies mainly description of the correlation between generally poor health, anemia, and poor outcomes in sepsis, rather than a direct causative correlation between low hemoglobin and mortality in sepsis.

Although it is known that lower hemoglobin concentration in the context of increased oxygen demand as is frequently the case in sepsis may play a negative role and augment organ dysfunction, forced elevation of hemoglobin concentration by blood transfusions does not necessarily mean improvement in organ function [17]. Hence, the role of blood transfusion in preservation of organ function in sepsis is still debated. In order to characterize blood transfusion practice in our cohort, we compared the characteristics of patients who received blood transfusions to those who did not. 8 % of the cohort received blood transfusions. These patients were significantly older by an average of 4 years with higher rates of malignancies and terminal diseases (Table 2). Bleeding was documented in 20 % of the transfused patients, while in the remaining cases it seems that hemoglobin concentration was the indication for transfusion, with a significantly higher percent of patients with moderately and severely reduced hemoglobin levels on admission. Although older, patients that received blood transfusions had no significant differences in the rate of acute events of myocardial infarction or respiratory failure as may be expected; neither had they had more co-morbid cardiovascular diseases. A similar finding regarding the lower hemoglobin level as the main difference between transfused and non-transfused patients was shown in a previous study that investigated blood transfusion in patients with septic shock treated in an ICU [18]. An important consideration related to blood transfusion is the complications that are described in about 20 % of cases [19]. In our cohort, the rate of complications was low (about 3 %) mostly non fatal hemolytic reactions. As was mentioned before, the decision of giving a blood transfusion for any patient in our hospital is based on the treating physician consideration rather than a specific transfusion protocol.

In our cohort, although transfused patients had a significantly higher in hospital mortality and worse longtime survival, blood transfusion itself was not found to be an independent risk factor for these outcomes, but it rather seems to be a marker of a disease severity as the transfused patients were older, more debilitated and endured terminal diseases more frequently. Previous studies investigating the correlation between blood transfusion in sepsis and mortality found conflicting results. While as observed in the present study, in some studies blood transfusion was not found to correlate with mortality [18, 20–22] other studies showed an improved outcome for patients who received blood transfusion [23, 24]. It is important to emphasize that these studies focused mainly on critically ill patients with septic shock in the setting of ICU, while our cohort included patients with a wide range of disease severity of sepsis and older patients with high proportion of chronic diseases, representing the majority of patients with sepsis at hospitals.

Whether or not increasing hemoglobin level can improve the prognosis in sepsis is still an unresolved question as is the controversial issue of hemoglobin concentration threshold for transfusion and the desired optimal level that should be targeted. A significant progression in this field had been achieved in cases of septic shock, mainly due to a recent large, multicenter, randomized study that compared a hemoglobin level threshold of 9 g/dL vs. 7 g/dL for blood transfusion in patients with septic shock in the ICU and found no difference in 90 day mortality [25].

### Learning points

- Our study investigated an important population of patients with sepsis that is treated in general departments that differs significantly from those treated at ICUs and seems to represents better the majority of patients with sepsis in hospitals.
- Our study shows the beneficial effect of higher hemoglobin levels on presentation and during early stage of sepsis, yet due to the observational design of the current study the results do not support conclusions about optimal hemoglobin concentration and the optimal strategy for blood transfusions in septic patients in the setting of IM departments.

## Conclusions

Our study shows the beneficial effect of higher hemoglobin levels on presentation and during early stage of sepsis. Currently, most physicians adopt a “restrictive” strategy of blood transfusion (transfuse when hemoglobin level < 7 g/dL) as recommended, except in acute hemorrhage or in patients with acute myocardial ischemia where a more liberal strategy is the usual practice [26].

Still, our study is a descriptive one that demonstrates an association between low hemoglobin levels and worsened outcome. Whether an interventional approach targeting higher hemoglobin levels can improve the outcome of patients with sepsis remains to be determined in future prospective studies.

## Abbreviations

ANOVA: Analysis of variance
CHF: Congestive heart failure
CI: Confidence interval
CKD: Chronic kidney disease
DIC: Disseminated coagulation disorder
HGB: Hemoglobin
HR: Hazard ratio
ICU: Intensive care unit
IHD: Ischemic heart disease
IM: Internal medicine
LTCF: Long- term care facility
OR: Odds ratio
RBC: Red blood cell
SSTI: Skin and soft tissue infections
UTI: Urinary tract infection
